# Cellular 5′-3′ mRNA Exoribonuclease XRN1 Inhibits Interferon Beta Activation and Facilitates Influenza A Virus Replication

**DOI:** 10.1128/mBio.00945-21

**Published:** 2021-07-27

**Authors:** Yen-Chin Liu, Bobo Wing-Yee Mok, Pui Wang, Rei-Lin Kuo, Honglin Chen, Shin-Ru Shih

**Affiliations:** a State Key Laboratory for Emerging Infectious Diseases, Department of Microbiology, The University of Hong Kong, Hong Kong SAR, People’s Republic of China; b Collaborative Innovation Center for Diagnosis and Treatment of Infectious Diseases, The University of Hong Kong, Hong Kong SAR, People’s Republic of China; c Research Center for Emerging Viral Infection, College of Medicine, Chang Gung University, Taoyuan, Taiwan; d Graduate Institute of Biomedical Sciences, College of Medicine, Chang Gung University, Taoyuan, Taiwan; e Department of Medical Biotechnology and Laboratory Science, College of Medicine, Chang Gung University, Taoyuan, Taiwan; f Department of Laboratory Medicine, Linkou Chang Gung Memorial Hospital, Taoyuan, Taiwan; g Research Center for Chinese Herbal Medicine, Research Center for Food and Cosmetic Safety, and Graduate Institute of Health Industry Technology, College of Human Ecology, Chang Gung University of Science and Technology, Taoyuan, Taiwan; The Peter Doherty Institute for Infection and Immunity

**Keywords:** influenza virus, 5′-3′ mRNA degradation, XRN1, nonstructural protein 1, NS1, interferon beta, IFN-β, viral replication, innate immune response

## Abstract

Cellular 5′-3′ exoribonuclease 1 (XRN1) is best known for its role as a decay factor, which by degrading 5′ monophosphate RNA after the decapping of DCP2 in P-bodies (PBs) in *Drosophila*, yeast, and mammals. XRN1 has been shown to degrade host antiviral mRNAs following the influenza A virus (IAV) PA-X-mediated exonucleolytic cleavage processes. However, the mechanistic details of how XRN1 facilitates influenza A virus replication remain unclear. In this study, we discovered that XRN1 and nonstructural protein 1 (NS1) of IAV are directly associated and colocalize in the PBs. Moreover, XRN1 downregulation impaired viral replication while the viral titers were significantly increased in cells overexpressing XRN1, which suggest that XRN1 is a positive regulator in IAV life cycle. We further demonstrated that the IAV growth curve could be suppressed by adenosine 3′,5′-bisphosphate (pAp) treatment, an inhibitor of XRN1. In virus-infected *XRN1* knockout cells, the phosphorylated interferon regulatory factor 3 (p-IRF3) protein, interferon beta (*IFN-β*) mRNA, and interferon-stimulated genes (ISGs) were significantly increased, resulting in the enhancement of the host innate immune response and suppression of viral protein production. Our data suggest a novel mechanism by which the IAV hijacks the cellular XRN1 to suppress the host innate immune response and to facilitate viral replication.

## INTRODUCTION

Influenza A virus (IAV) causes seasonal epidemics and occasional pandemics, leading to considerable mobility and mortality worldwide. The World Health Organization (WHO) estimates that annual epidemics of influenza result in ∼1 billion infections, 3 to 5 million cases of severe illness, and 300,000 to 500,000 deaths (1–3). The emergence of a novel influenza virus remains a worldwide threat, and effective antiviral drugs and universal influenza virus vaccines to protect against new viral pandemics are lacking (4–6). Therefore, research focusing on virus-host interactions may provide valuable insights into the identification of new therapeutic targets for influenza (7).

The IAV nonstructural protein 1 (NS1), which is encoded by the NS gene segment and expressed in the cytoplasm and nuclei of infected cells, has multiple functions and plays a central role in regulating viral replication mechanisms, inhibiting host innate/adaptive immune responses, and enhancing viral mRNA translation and regulation of virus replication through NS1-host RNA and protein interactions (8–11). NS1 downregulates interferon (IFN) production by inhibiting interferon regulatory factor 3 (IRF3) activity and IFN transcription, as well as through suppression of IFN pre-mRNA processing (9). The interaction between NS1 and the E3 ligase tripartite motif-containing protein 25 (TRIM25), which results in the suppressed ubiquitylation and activation of retinoic acid-inducible gene I (RIG-I) (12, 13). A mutation in position 171 of the NS1 protein decreases the expression of IFN and IFN-stimulated genes (ISGs) (14). The RNA-binding domain (RBD) of the NS1 protein is required for interaction with host splicing regulator SF2 in the nucleus that modulates splicing of NS mRNAs during influenza virus replication (15). Our previously reported results suggest that NS1 interacts with cellular P-bodies (PBs) and stress granules (SGs) through RNA-associated protein 55 (RAP55) during H5N1 infection (16). In this research, we further discovered that IAV NS1 can directly associate with 5′-3′ exoribonuclease XRN1, which is the critical factor for degrading 5′-3′ mRNA in PBs.

PBs functions as a site of mRNA decay, specifically that involving the 5′-to-3′ mRNA decay pathway, which carries out cytoplasmic mRNA degradation in *Drosophila*, yeast, and mammals (17–19). The 5′-to-3′ mRNA decay process occurs following 3′ deadenylation, consisting of mRNA decapped by mRNA-decapping enzyme 2 (DCP2), and mRNA-decapping enzyme 1A (DCP1A). EDC4 acts as a scaffold to provide binding sites for DCP1A trimers at its N-terminal WD40 domain and for DCP2 and XRN1 at its C-terminal α-helical domain. The decapped and 5′ monophosphate RNA is then degraded from the 5′ end by exoribonuclease XRN1 (20–23).

The antiviral activity of XRN1 and DCP1/2 aggregation against cytoplasmic RNA viruses, such as Newcastle disease virus (NDV) and encephalomyocarditis virus (EMCV), effectively prevented cell death (24). In infection with flaviviruses such as Zika, dengue, and West Nile virus, XRN1 is stalled and blocked from continuing degradation at the site of the viral mRNA 3′ untranslated region (3′-UTR) due to the presence of pseudoknots or stem loops, which are referred as XRN1-resistant structures (xrRNAs). The secondary structure of these viral mRNAs inhibits XRN1 function, which prevents the degradation of viral mRNA and leads to subgenomic flavivirus RNA (sfRNA) production. XRN1 is stalled at the 5′-UTR due to binding of hsa-miR-122 at the seed sequence site and the presence of stem loops during *Flaviviridae* hepatitis C virus (HCV) infection (25–28). However, XRN1 can regulate double-stranded RNA (dsRNA) accumulation and dsRNA-responsive innate immune effectors in vaccinia virus (VacV)-infected cells (29). In addition, XRN1 is also involved in completing the degradation of host antiviral mRNAs following virus-induced endonucleolytic cleavage processes, thereby suppressing cellular gene expression and evading host antiviral innate immune defenses in herpesvirus (HHV), severe acute respiratory syndrome (SARS) coronavirus (SCoV), and IAV infection (30, 31). Here, we further found that the phosphorylated IRF3 (p-IRF3) significantly increased in IAV-infected XRN1-depleted cells. Thus, we think that XRN1 is also involved in the upstream events of IFN gene transcription.

The three members of the RIG-I-like receptor (RLR) family that recognize RNA and are IFN inducible, are retinoic acid-inducible gene I (RIG-I), melanoma differentiation factor 5 (MDA5), and laboratory of genetics and physiology 2 (LGP-2) (32). RIG-I and MDA5 distinguish different RNA viruses and are critical for type I interferon responses. RIG-I preferentially senses 5′ triphosphorylated ssRNA (pppRNA) and short dsRNA whereas MDA5 recognizes long dsRNA (33, 34). In addition, RIG-I is a key mediator of interferon beta (IFN-β) production in response to IAV RNA viruses and is activated by viral genomic single-stranded RNA (ssRNA) bearing 5′-phosphates (35), whereas MDA5 is a significant contributor to host defense against influenza A virus (36). Both RIG-I and MDA5 have the N-terminal tandem caspase activation recruitment domain (2CARD), which can interact with mitochondrial antiviral signaling protein (MAVS) on mitochondria or peroxisomes to activate the downstream TBK1 induction and IRF3 phosphorylation. The phosphorylated IRF3 forms a dimer and translocates to the nucleus, activating the transcription of *IFN-β*, and then triggering the downstream gene expression of ISGs with antiviral functions (37–39). Our findings showed that host XRN1 expression was positively correlated with IAV replication and negatively associated with the expression of immune-related genes, resulting in the shutdown of host *IFN-β* expression and advancement of viral replication, thus contributing to the pathogenesis of viral infection.

## RESULTS

### NS1 directly associates with the host XRN1 in the PBs.

**FIG 1:**
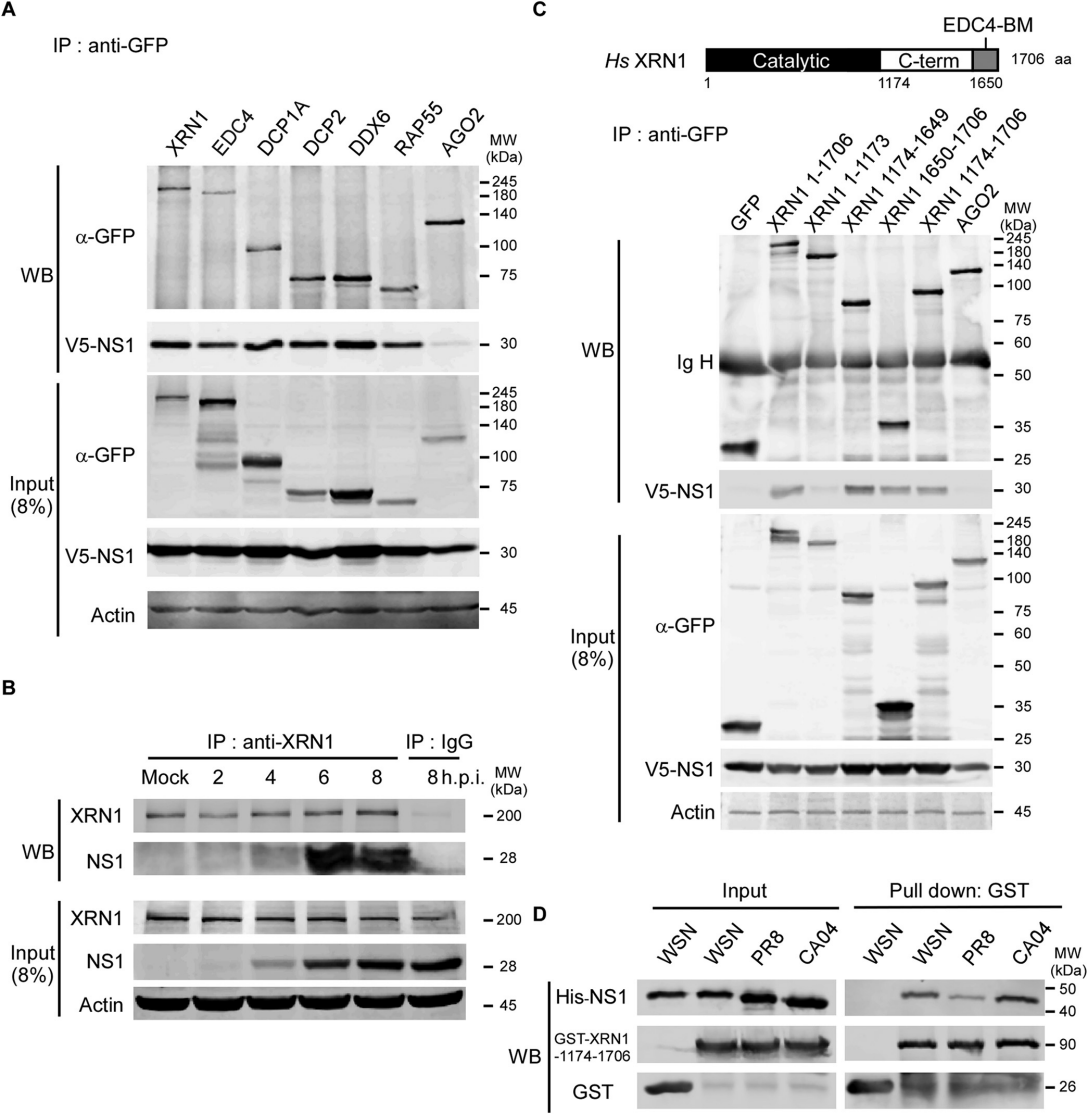
NS1 directly associates with the cytoplasmic protein XRN1. (A) NS1 interacts with the components of 5′-to-3′ mRNA decay factors in PBs, including XRN1, EDC4, DCP1A, DCP2, DDX6, and RAP55. HEK293T cells were transfected with plasmids encoding WSN V5-NS1, as well as cellular GFP-XRN1, EDC4, DCP1A, DDX6, RAP55, and AGO2. At 48 h after transfection, the lysates were treated with RNase A (10 μg/ml) and immunoprecipitated (IP) with antibodies against GFP (α-GFP). The components of P-bodies that interacted with NS1 were detected by Western blotting (WB) using an antibody against V5. The input samples were verified in the presence of NS1 and the components of the P-bodies in the lysates. Actin served as an internal control. The positions of molecular weight (MW) markers (in kilodaltons) are shown to the right of the gel. (B) Endogenous XRN1 associates with NS1 in virus-infected cells. The lysates harvested from A549 cells that were mock infected or infected with WSN at an MOI of 2 at various times postinfection (2 to 8 h.p.i.) were treated with RNase A (10 μg/ml) and immunoprecipitated using an anti-XRN1 antibody and an anti-IgG antibody as a negative control. Then, the interacting NS1 constructs were detected by a WB assay. (C) NS1 associates with the C-terminal region of XRN1 containing the C-terminal domain (amino acids 1174 to 1649 [aa 1174–1649]) and EDC4-BM domain (aa 1650–1706). The functional domain architecture of human (Homo sapiens [Hs]) XRN1 is shown at the top of panel C. HEK293T cells were transfected with plasmids encoding V5-NS1, various truncated forms of GFP-XRN1, and GFP. At 48 h after transfection, the lysates were treated with RNase A (10 μg/ml) and immunoprecipitated with antibodies against GFP. The bound NS1 was detected by WB using an antibody against V5. GFP served as a negative control. (D) The association between XRN1 and WSN NS1 is direct without RNA or protein intermediary. In the *in vitro* GST pulldown and WB assay, a total of 5 μg of bacterially purified His^+^-NS1 from WSN, PR8, or CA04 was mixed with the GST-XRN1-C-terminal region (aa 1174–1706) fusion protein, followed by WB with anti-His and anti-GST.

We previously reported that the NS1 protein of IAV interacts with cellular PBs and SGs through RAP55 during viral infection (16). In this study, we further confirmed that NS1 can interact with proteins of the 5′-to-3′ mRNA degradation pathway, including XRN1, EDC4, DCP1A, DCP2, DDX6, and RAP55, but not with AGO2, as it does not belong to the 5′-to-3′ mRNA degradation complex. However, AGO2 is present in the PBs and can stablish AGO-microRNA (miRNA) interactions and target the mRNAs of these structures (40). These interactions without RNA intermediary were detected by overexpressing multiple GFP fusion proteins as well as V5-NS1 in HEK293T cells and analyzing the cell lysates following RNase A treatment using coimmunoprecipitation (Co-IP) and Western blotting (WB) assays. The input samples, whole-cell lysates at 8% concentration, were analyzed to evaluate the presence of NS1 and P-body components (Fig. 1A). Therefore, XRN1, which is a critical 5′-to-3′ mRNA-degrading enzyme in the PBs, was selected for further studies. The interaction between XRN1 and NS1 was further verified in WSN-infected A549 cell lysates following RNase A treatment by Co-IP and WB assays with antibodies against endogenous XRN1 and viral NS1, respectively. The results of these assays suggested that endogenous XRN1 interacts with WSN NS1 between 4 to 8 h postinfection (h.p.i.), without the presence of an RNA intermediary (Fig. 1B).

There are three functional domains within cellular XRN1, including the catalytic domain (amino acids 1 to 1173 [aa 1–1173]), C-terminal domain (aa 1174–1649), and EDC4-binding motif (EDC4-BM) domain (aa 1650–1706). To identify the interacting domains of XRN1 and NS1, we constructed tags that were fused with GFP, for various truncated forms of XRN1, and the fragments of XRN1 were cloned separately from each functional domain. Plasmids were transfected into HEK293T cells, followed by anti-GFP immunoprecipitation (IP) and WB assays. This mapping study revealed that the full-length XRN1 and the C-terminal region (aa 1174–1706) containing the C-terminal and EDC4-BM domains of XRN1 interacted with full-length NS1 in an RNA binding-independent manner (Fig. 1C). We next used an *in vitro* pulldown assay to assay whether the interaction between XRN1 and NS1 was direct. Purified His^+^-NS1 from various viruses WSN, PR8, CA04, and the GST-XRN1-C-terminal region fusion proteins were mixed and subjected to glutathione *S*-transferase (GST) pulldown and WB assays. The result revealed that the NS1 protein directly associates with the C-terminal region of XRN1 (Fig. 1D). These results suggest that NS1 directly associates with the C-terminal region of cellular XRN1 in the PBs.

### NS1 assembles and colocalizes with XRN1 in the cellular cytoplasm at 6 and 8 h.p.i.

**FIG 2:**
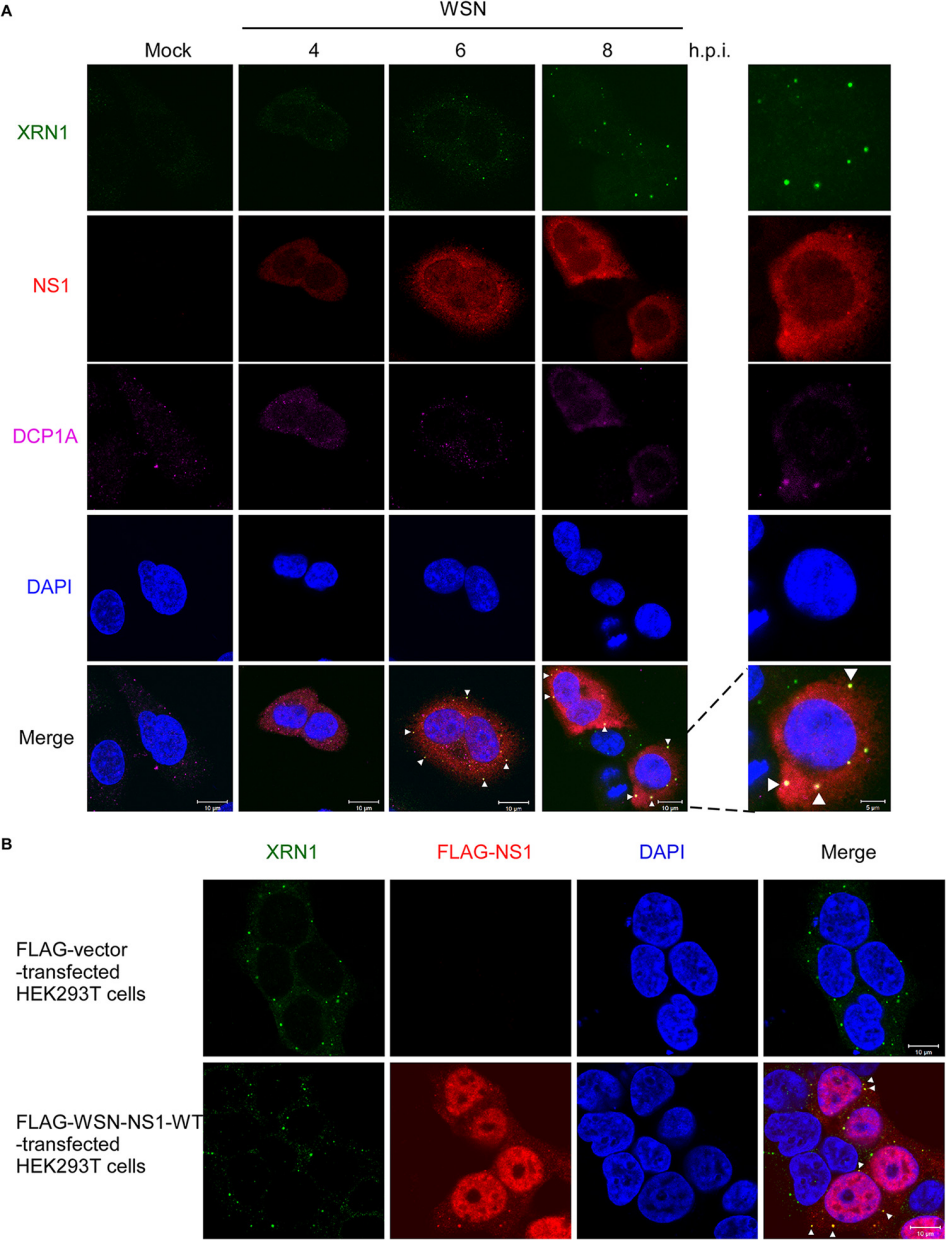
WSN NS1 and XRN1 colocalize in the cytosol at 6 and 8 h.p.i. (A) The WSN NS1-XRN1 association is localized in the cytosol at 6 and 8 h.p.i. Mock-infected A549 cells or A549 cells infected with WSN at an MOI of 2 were fixed and stained using antibodies against XRN1 (green color), WSN NS1 (red color), and DCP1A (purple color) at 4, 6, and 8 h.p.i. The nuclei of A549 cells were stained with DAPI (blue color), and the merged images show the NS1 and XRN1 immunofluorescence signals. All immunofluorescence images were detected by confocal microscopy. Bars, 5 and 10 μm. (B) Endogenous XRN1 colocalizes with FLAG-WSN-NS1 in the cytoplasm. HEK293T cells were transfected with a plasmid expressing FLAG-NS1 for 48 h and then stained using antibodies against FLAG (red color) and against endogenous XRN1 (green color). The nuclei were stained with DAPI dye (blue color). Bars, 10 μm.

We further examined the distribution of cellular XRN1 and viral NS1 in virus-infected cells. The localization of NS1 and XRN1 in A549 cells during a time course of WSN infection was studied using anti-XRN1 (green color), anti-NS1 (red color), and anti-DCP1A (purple color) antibodies in an immunofluorescence assay (IFA) by confocal microscopy. DCP1A was used as a biomarker for the PBs (Fig. 2A). The confocal images showed that XRN1, NS1, and DCP1A were mostly located in the cytoplasm at 4, 6, and 8 h.p.i. In addition, XRN1 and NS1 accumulation and colocalization were detected in the merged images at 6 and 8 h.p.i as yellow dots (Fig. 2A, white arrowheads).

HEK293T cells were then transfected with FLAG-tagged NS1 (FLAG-NS1), and the resulting fluorescence was detected with an anti-FLAG antibody (green color) by IFA and confocal microscopy. The overexpressed FLAG-NS1 with the nuclear localization sequence was partially expressed in the nucleus, whereas the FLAG-NS1 was also expressed and accumulated with the endogenous XRN1 in the cytoplasm (Fig. 2B). Colocalization of endogenous XRN1 and FLAG-NS1 was detected in the cytoplasm of HEK293T cells, it can be observed as yellow dots in the merged images of Fig. 2B (white arrowheads). These results indicated that cellular endogenous XRN1 colocalized with the IAV NS1 in infected cells and then assembled and accumulated in the cytoplasm to form the observed dots from 6 to 8 h.p.i.

### XRN1 contributes to IAV replication in human cells.

**FIG 3:**
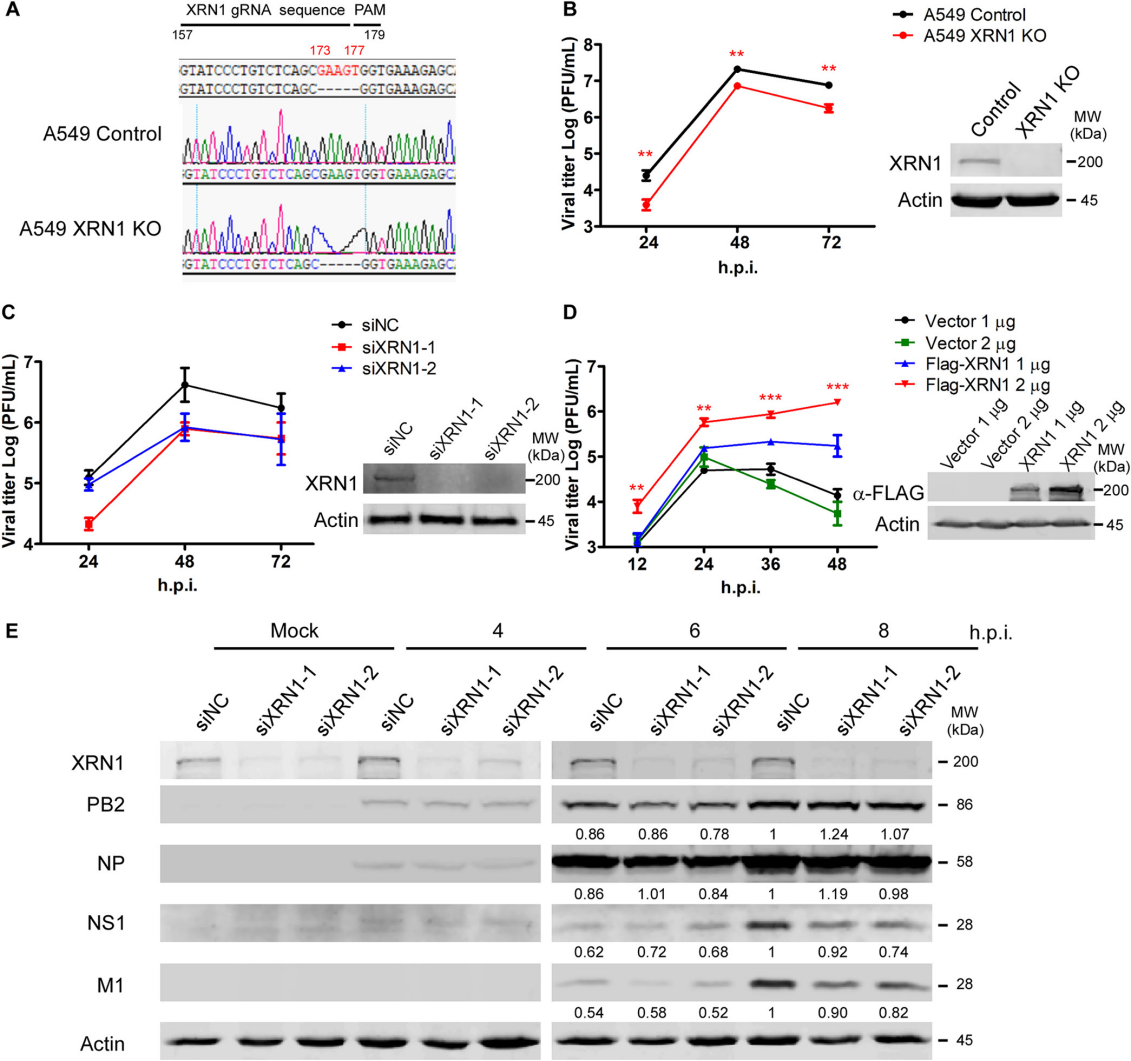
XRN1 functions as a positive regulator of IAV replication. (A) The genomic DNA sequence of human A549 control cells and *XRN1* knockout cells. There are five genomic nucleotides (GAAGT, 173 to 177 bp) deleted in *XRN1* knockout A549 cells. PAM, protospacer adjacent motif. (B) The viral growth curve is decreased in *XRN1* knockout cells. The growth kinetics of WSN viruses were determined at an MOI of 0.01 for multicycle infections of human A549 control cells and *XRN1* knockout cells. The viral supernatants were harvested at 24, 48, and 72 h.p.i., and the viral titers were detected by plaque assay. Values are shown as means ± standard deviations (SD) (error bars) (*n* = 3). Statistical significance was analyzed using a *t* test and indicated as follows: **, *P* < 0.01. The XRN1 protein expression level was assessed by WB assay. (C) Viral titer is decreased in XRN1 knockdown cells. A549 cells were transfected with siRNA to knock down XRN1 for 72 h and then infected with WSN viruses at an MOI of 0.01 for multicycle infections. The viral titers were detected at 24, 48, and 72 h.p.i. by plaque assay, and the transfection efficiency was assessed by WB. The viral growth curve was constructed with data from two independent plaque assays (*n* = 2). (D) Overexpressed FLAG-full length-XRN1 protein in HEK293T cells enhances the viral growth curve. HEK293T cells were treated with various quantities of FLAG-tagged vector (FLAG-Vector) or FLAG-XRN1 for 48 h, then infected with WSN viruses at an MOI of 0.01. At 24, 48, and 72 h.p.i., the viral titers for the multicycle infections were detected by plaque assay, and the transfection efficiency was assessed by WB. Two micrograms of overexpressed FLAG-XRN1 was compared with 2 μg of FLAG-Vector. . Values are shown as means ± standard deviations (SD) (error bars) (*n* = 3). Statistical significance was analyzed using a *t* test and indicated as follows: ***, *P* < 0.001; **, *P* < 0.01. (E) Viral NS1 and M1 proteins are reduced by XRN1 knockdown of A549 cells. A549 cells were transfected with siNC as a negative control and with XRN1-specific siRNA (siXRN1-1 and -2) on 3 days. After WSN infection at an MOI of 2 at 4, 6, and 8 h, the viral proteins were isolated and detected using specific antibodies. Virus protein levels were quantified with the AlphaEase FC software, and the values were normalized against actin expression.

We sought to investigate the impact of XRN1 on the replication of influenza A viruses in human cells. Therefore, we used the CRISPR-Cas9 system to generate a stable human A549 XRN1 knockout cell line. The *XRN1* guide RNA (gRNA) sequence (GTATCCCTGTCTCAGCGAAG) targeted the complementary sequence of *XRN1* genomic DNA from 157 to 176 bp before the protospacer adjacent motif (TGG, 177 to 179 bp). *XRN1* genomic DNA with a five-nucleotide deletion (GAAGT, 173 to 177 bp) was expressed in A549 XRN1 knockout (KO) cells as well as A549 control cells (Fig. 3A). A549 XRN1 KO cells were used for viral growth kinetic studies and infected at a multiplicity of infection (MOI) of 0.01 in a multicycle experiment. The viral titer of A549 XRN1 KO cells decreased significantly compared with control cells at 24 to 72 h postinfection. Thus, XRN1 functions as a positive regulator in the WSN-infected A549 cells (Fig. 3B). Moreover, A549 cells were treated with negative-control (NC) small interfering RNA (siRNA) and XRN1 siRNA (siXRN1-1, siXRN1-2) to knock down *XRN1*, and then, the cells were infected with WSN at an MOI of 0.01. Consistent with the *XRN1* knockout results, the growth curves of siXRN1-1 and siXRN1-2 showed a decrease compared with A549 cells in multicycle experiments (Fig. 3C). HEK293T cells were treated with the FLAG-tagged vector or FLAG-tagged XRN1 (FLAG-XRN1) plasmid as a negative control as well as with full-length XRN1 expressed, then infected with WSN in a time course of 12, 24, 36, and 48 h. The data revealed that overexpressed FLAG-XRN1 resulted in a significant increase in viral titer compared with the FLAG-tagged vector (Fig. 3D).

We also measured the expression levels of viral proteins in A549 XRN1 knockdown and control cells. The siNC, siXRN1-1, and siXRN1-2 were transfected into A549 cells, followed by WSN infection. Viral NS1 and M1 protein expression yielded a lower signal in siXRN1-1 and siXRN1-2 infected cells at 8 h.p.i. (Fig. 3E). Therefore, our findings suggest that cellular XRN1 is essential for positive regulation of viral growth curves and viral protein expression.

### IAV titers decrease after inhibition of cellular XRN1 with adenosine 3′, 5′-bisphosphate.

**FIG 4:**
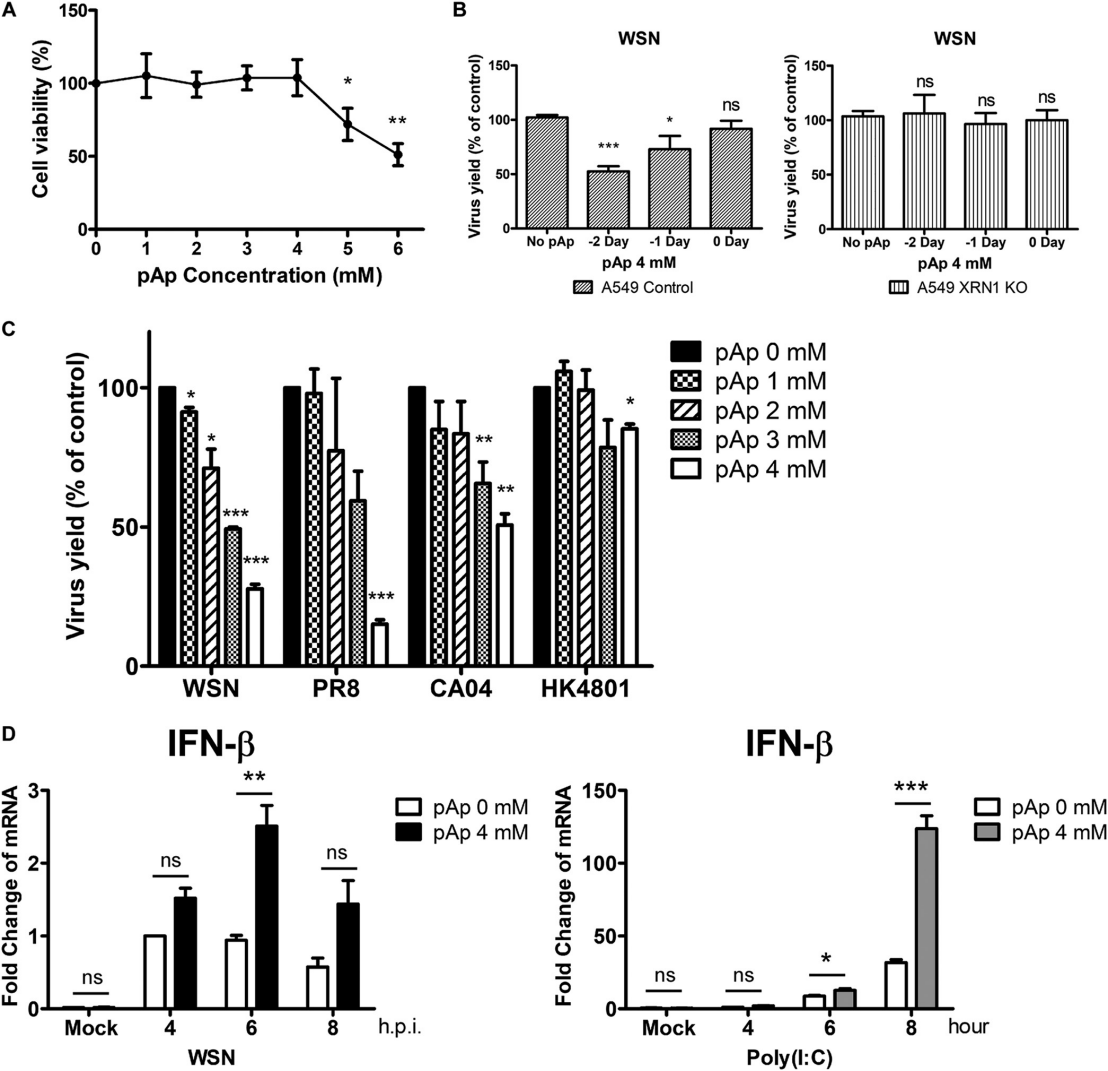
The pAp inhibits viral replication in a broad range of influenza virus strains. (A) Viability of A549 cells after pAp treatment. A549 cells were treated with pAp in a dose-dependent manner for 3 days. We then used MTT assay to measure the cellular viability and cytotoxicity. (B) The pAp disrupts viral replication most effectively for 2 days treatment before viral infection. A549 cells or XRN1 KO cells were pretreated with 4 mM pAp 2, 1, and 0 days before WSN infection at an MOI of 0.01. The viral supernatants were harvested, and viral titers were determined by plaque assay. (C) The pAp reduces the IAV replication in a dose-dependent manner. A594 cells were pretreated with pAp for 2 days and then infected with the influenza virus strains A/WSN/33 (H1N1), A/Puerto Rico/8/34 (H1N1), A/California/04/2009 (H1N1), and A/Hong Kong/4801/2014 (H3N2). Virus yields were determined by plaque assay and reported as a percentage of the value for untreated controls. All exhibited dose-dependent reductions in virus plaque formation after treatment with 1, 2, 3, and 4 mM pAp. (D) A549 cells were pretreated with 4 mM pAp for 2 days and infected with WSN for 4, 6, and 8 h (left panel) or treated with poly(I:C) during 4, 6, and 8 h (right panel). The mRNAs levels of *IFN-β* were detected by RT-qPCR. Data represent the means ± SD of three independent experiments. Statistical significance was determined by a *t* test and indicated as follows: ***, *P* < 0.001; **, *P* < 0.01; *, *P* < 0.05; ns, not significant.

Since previous studies have revealed that adenosine 3′,5′-bisphosphate (pAp) can inhibit the 5′-to-3′ exonuclease activity of XRN1 (41, 42), we tested whether pAp was able to affect IAV replication by blocking the exonuclease function of XRN1. First, we evaluated the viability and cytotoxicity of pAp in A549 cells with the 3-(4,5-dimethylthiazol-2-yl)-2,5-diphenyltetrazolium bromide (MTT) assay. We found that 1 to 4 mM pAp treatment did not affect cellular viability and that the 50% cytotoxic concentration (CC50) was 6.06 mM (Fig. 4A). Then, we performed a time of addition assay to assess the effect of pAp on influenza virus replication. A549 cells were pretreated with pAp (4 mM) 2, 1, or 0 days before infection with WSN strain at an MOI of 0.01, and the viral supernatant was harvested at 48-h time points postinfection. The results revealed that pAp inhibited virus yields more effectively when it was applied 2 days before virus infection. XRN1 KO cells were treated with pAp to evaluate the occurrence of nonspecific effects on viral replication in the absence of XRN1 (Fig. 4B). We pretreated A549 cells with 1, 2, 3, or 4 mM pAp for 2 days and infected them with WSN (H1N1), PR8 (H1N1), CA04 (H1N1), or HK4801 (H3N2) during 48 h, and after incubation, the culture supernatants were harvested for plaque assay analysis. All conditions exhibited dose-dependent reductions in virus plaque formation after treatment with 3 and 4 mM concentrations of the XRN1 inhibitor pAp (Fig. 4C), indicating that the XRN1 inhibitor pAp can inhibit the replication of different influenza strains. In addition, A549 cells that had been pretreated with 4 mM pAp for 2 days were infected with WSN or exposed to synthetic polyinosinic-polycytidylic acid [poly(I:C)] dsRNA for 4, 6, and 8 h, and mRNA expression of *IFN-β* was detected by reverse transcription-quantitative PCR (RT-qPCR) (Fig. 4D). *IFN-β* levels significantly increased in pAp-treated A549 cells infected with WSN or exposed to poly(I:C), suggesting that pAp directly affects *IFN-β* induction, and not WSN infection *per se*. These results further demonstrate that loss of XRN1 activity in the pAp-treated cells results in the inhibition of IAV replication and upregulation of *IFN-β* mRNA levels.

### XRN1 contributes to effective IAV inhibition of host immune responses.

**FIG 5:**
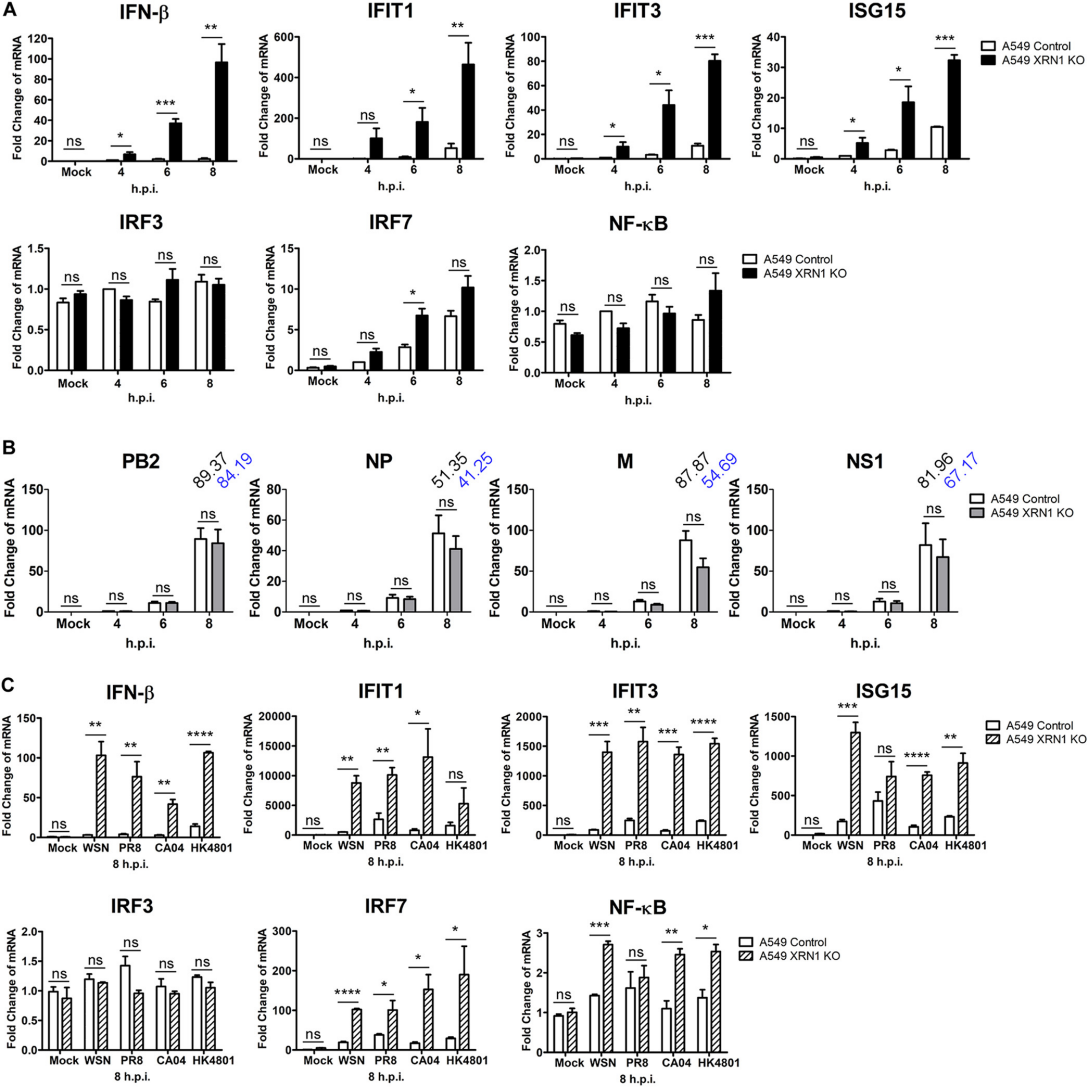
XRN1 contributes to reducing the innate immune system. (A) Virus heavily elicits cellular mRNAs of the innate immune response in *XRN1* knockout A549 cells. RNAs were isolated from WSN-infected control and *XRN1* knockout A549 cells at 4 to 8 h.p.i. and evaluated using specific primers for *IFN-β*, *IFIT1*, *IFIT3*, *ISG15*, *IRF3*, *IRF7*, and *NF-κB* by RT-qPCR. All assays were performed and repeated three times. The fold changes in the amount of mRNA were calculated. (B) The level of viral mRNA in WSN-infected A549 control cells and *XRN1* knockout A549 cells. RNAs were isolated from WSN-infected control and *XRN1* knockout A549 cells at 4, 6, and 8 h and evaluated with specific primers for viral PB2, NP, M, and NS1 by RT-qPCR. The fold changes in the amount of mRNA were calculated. (C) There are significantly increased levels of *IFN-β*, *IFIT1*, *IFIT3*, and *ISG15* mRNA in *XRN1* KO cells infected with certain IAV constructs. The A549 control and *XRN1* knockout cells were infected with various IAV constructs, including A/WSN/33 (H1N1), A/Puerto Rico/8/34 (H1N1), A/California/04/2009 (H1N1), and A/Hong Kong/4801/2014 (H3N2), at an MOI of 2 for 8 h. Levels of cellular mRNAs were measured by RT-qPCR. Data represent means plus standard errors of the means of three independent experiments. Statistical significance was determined by a *t* test and indicated as follows: ***, *P* < 0.001; **, *P* < 0.01; *, *P* < 0.05; ns, not significant.

Considering that XRN1 inhibitor pAp contributes to the induction and upregulation of IFN-β, we further investigated the effect of *XRN1* KO cells on the induction of IFN and ISGs. A549 control and *XRN1* KO cells were infected with WSN at an MOI of 2 for 4, 6, and 8 h, and the mRNA expression levels of different cytokines, including *IFN-β*, *IFIT1*, *IFIT3*, *ISG15*, *IRF3*, *IRF7*, and *NF-κB* were measured by RT-qPCR. The A549 cells lacking the XRN1 protein showed increased *IFN-β*, *IFIT1*, *IFIT3*, and *ISG15* expression compared with the A549 control cells at 4 to 8 h after WSN infection (Fig. 5A). We found that mock-infected cells showed the lowest levels of *IFN* and *ISG* expression and that *IFN* and *ISG* basal levels did not increase in XRN1 knockout cells or pAp-treated cells (Fig. 5A and 4D). In addition, we transfected HEK293T cells with the FLAG-XRN1 plasmid and observed that XRN1 overexpression repressed the expression of innate response-related genes, such as *IFNB*, *IFIT1*, *IFIT3*, and *ISG15* in early stages of infection, 4 to 6 h.p.i. Moreover, at 8 h.p.i., the expression of these genes, in vector- or FLAG-XRN1-transfected cells was inhibited due to viral resistance (see Fig. S2A and B in the supplemental material).

10.1128/mBio.00945-21.3 FIG S2 The overexpressed XRN1 proteins in WSN-infected cells inhibit innate immune response. (A) HEK293T cells were transfected with 2 μg of FLAG-XRN1 and then infected with WSN virus at an MOI of 2. After 4, 6, and 8 h postinfection (h.p.i.), XRN1 protein expression was detected by Western blotting using an anti-XRN1 antibody. (B) FLAG-XRN1 effect on *IFN-β*, *IFIT1*, *IFIT3*, and *ISG15* mRNA expression at 4 and 6 h after WSN infection. Data represent the means ± SD of three independent experiments. Statistical significance was determined by a *t* test. **, *P* < 0.01; *, *P* < 0.05; ns, not significant. Download FIG S2, TIF file, 0.5 MB. Copyright © 2021 Liu et al. 2021 Liu et al. https://creativecommons.org/licenses/by/4.0/ This content is distributed under the terms of the Creative Commons Attribution 4.0 International license.

We then investigated whether the increased mRNA expression levels of host innate immune cytokines were influenced by the virus. We detected the viral mRNA expression levels of PB2, NP, M, and NS1 in WSN-infected A549 control and *XRN1* KO cells at 4, 6, and 8 h. The levels of viral PB2, NP, M, and NS1 mRNA in A549 XRN1 KO cells were not significantly different than those in A549 control cells after statistical analysis, but the fold change of M mRNA decreased from 87.87- to 54.69-fold, and that of NS1 mRNA decreased from 81.96- to 67.17-fold in XRN1 KO cells at 8 h postinfection (Fig. 5B). We used another A549 *XRN1* knockout cell line as well as a control cell line, which were gifts from the NIH (43). We obtained consistent results when using the NIH A549 control and NIH A549 *XRN1* KO cell lines, which showed that the levels of mRNAs of the innate immune response were increased (see Fig. S1A to C in the supplemental material). There was higher expression of *IFN-β*, *IFIT1*, *IFIT3*, *ISG15*, and *IRF7* mRNA in A549 *XRN1* KO cells after 8 h of infection with various virus strains, including WSN (H1N1), PR8 (H1N1), CA04 (H1N1), and HK4801 (H3N2). However, the mRNA levels of *IRF3* and *NF-κB* were similar to those observed in the control cells (Fig. 5C). We discovered that *IFN-β*, *IFIT1*, *IFIT3*, and *ISG15* mRNA expression is upregulated in XRN1-KO cells infected with various IAV strains.

10.1128/mBio.00945-21.2 FIG S1 The *IFN* and *ISG* mRNA increase in NIH A549 XRN1 KO cells upon WSN infection. (A) RNAs were isolated from WSN-infected NIH A549 control cells and NIH A549 XRN1 KO cells at 4 to 8 h.p.i. and evaluated using specific primers for *IFN-β*, *IFIT1*, *IFIT3*, *ISG15*, *IRF3*, *IRF7*, and *NF-κB* by RT-qPCR. The fold changes in the amount of mRNA were calculated. Statistical significance was determined by a *t* test. ****, *P* < 0.0001; ***, *P* < 0.001; **, *P* < 0.01; *, *P* < 0.05; ns, not significant. (B) The viral RNAs were isolated from WSN-infected control and NIH A549 XRN1 knockout cells at 4, 6, and 8 h and evaluated with specific primers for viral PB2, NP, M, and NS1 by RT-qPCR. The fold changes in the amount of mRNA were calculated. Statistical significance was determined by a *t* test. ns, not significant. (C) The protein levels in NIH A549 control and NIH A549 XRN1 KO cells were identified with specific antibodies for XRN1 and actin by WB assay. Download FIG S1, TIF file, 0.4 MB. Copyright © 2021 Liu et al. 2021 Liu et al. https://creativecommons.org/licenses/by/4.0/ This content is distributed under the terms of the Creative Commons Attribution 4.0 International license.

### RIG-I-mediated signaling remains functional in XRN1-KO cells.

**FIG 6:**
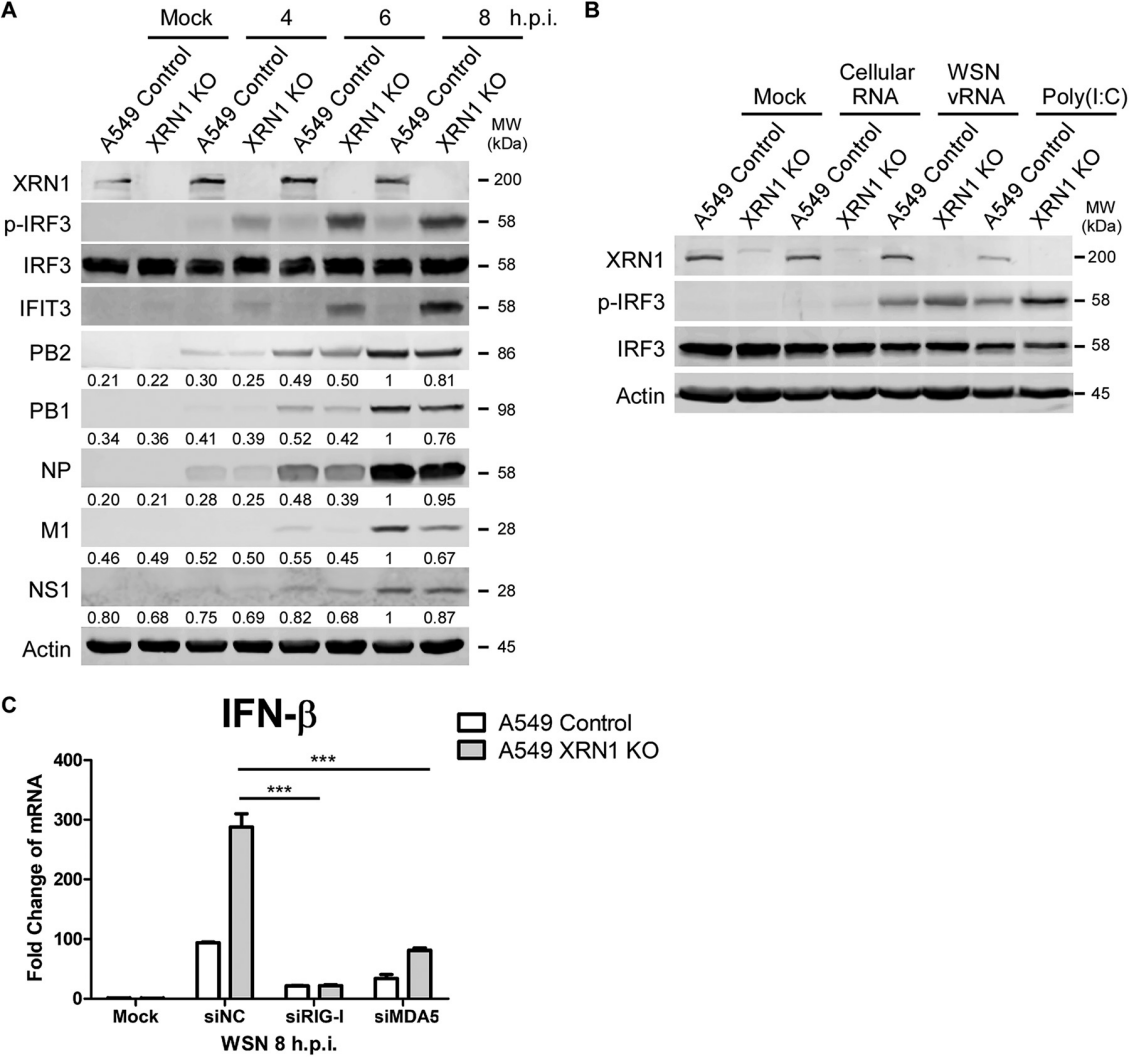
Cellular XRN1 suppresses IAV vRNA-induced IRF3 activation and IFN-β induction through RIG-I-mediated signaling pathway. (A) Host antiviral proteins activate and increase in A549 *XRN1* knockout WSN-infected cells. Cellular and viral proteins were harvested from WSN-infected control and *XRN1* knockout A549 cells at 4 to 8 h.p.i. and identified with the specific antibody for XRN1, p-IRF3, IRF3, IFIT3, PB2, PB1, NP, M, NS1, and actin by WB assay. Virus protein levels were quantified with the AlphaEase FC software, and the values were normalized against actin expression. (B) XRN1 has an impact on the influenza A virus vRNA-induced IRF3 activity and IFN-β induction. A549 control or XRN1-KO cells were transfected with 2 μg of total cellular RNAs, 2 μg vRNAs, and 1 μg poly(I:C) using Lipofectamine 2000 for 8 h. The cell lysates were harvested, and proteins were identified by WB assay. (C) A549 control and XRN1 KO cells were transfected with siRNA against RIG-I or MDA5 for 48 h, followed by infection with WSN for 8 h. The IFN-β mRNA levels were detected through RT-qPCR. Statistical significance was determined by a *t* test and indicated as follows: ***, *P* < 0.001.

Since the expression of *IFN-β* and downstream ISG genes was higher in XRN1-deficient cells than in A549 control cells after IAV infection, we now investigated whether XRN1 plays a role in the early steps of type I IFN (IFN-I) signaling cascade. We measured the protein levels of the upstream protein, phosphorylated IRF3 (p-IRF3), which is a critical transcription factor promoting type I interferon expression. Significant upregulation was observed for p-IRF3 in WSN-infected A549 *XRN1* KO cells compared with A549 control cells (Fig. 6A). To determine whether XRN1 affected IRF3 phosphorylation and IFN-β induction in IAV viral RNA (vRNA)-induced type I interferon immune system, the total WSN vRNA from supernatant of infected cells was prepared and transfected in XRN1-KO cells. The XRN1-KO cells were transfected with cellular RNA as a negative control, whereas poly(I:C), a synthetic analog of dsRNA, was used as a RIG-I and MDA5 agonist. The results showed that IRF3 was activated by WSN vRNA and that p-IRF3 was increased in WSN vRNA-transfected XRN1-KO cells compared to A549 controls, suggesting that XRN1 inhibits the vRNA-induced IRF3 activation (Fig. 6B). It is known that the viral RNA sensors RIG-I and MDA5 are key mediators for IFN-β production in response to RNA viruses. To verify whether RIG-I and MDA5 are still functional in XRN1-KO cells infected with IAV, *IFN-β* mRNA levels were quantified in the XRN1-KO cells knocked down for RIG-I and MDA5 endogenous expression, respectively. We found that knockdown of RIG-I or MDA5 in XRN1-depleted cells does not induce IFN-β mRNA production during IAV infection (Fig. 6C). These results suggest that IAV hijacks the cellular XRN1 to suppress host type I IFN signaling pathway.

### The coexistence of IAV NS1 and host XRN1 enhances the efficiency in inhibiting the antiviral immune response.

**FIG 7:**
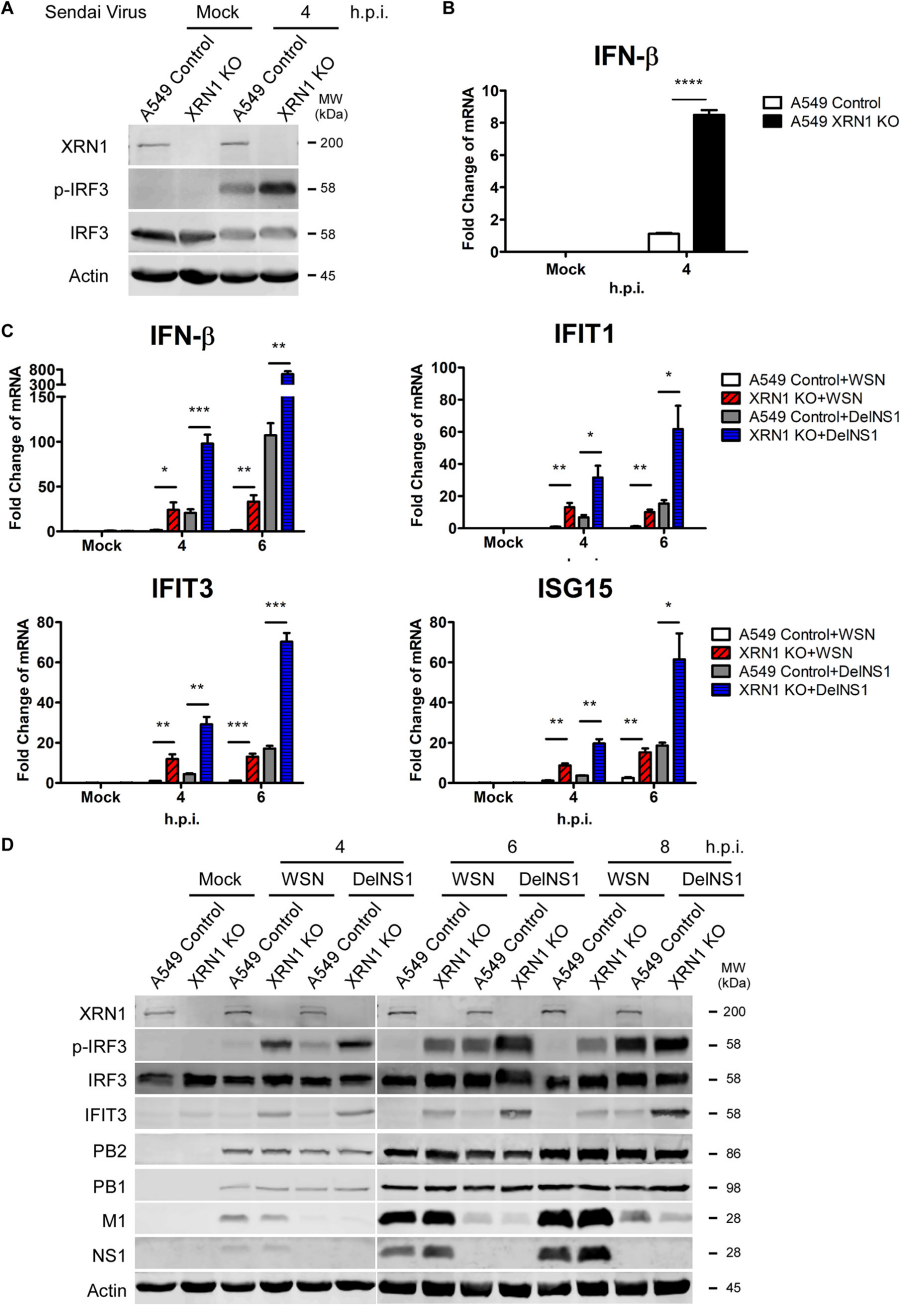
The coexistence of IAV NS1 and host XRN1 enhances the efficiency in blocking type I IFN response. (A) The Sendai virus also induces IRF3 activity and a higher expression ratio in *XRN1* KO cells compared with A549 control cells. Cellular and viral proteins were harvested from Sendai virus-infected A549 control and *XRN1* KO cells at 4 h.p.i. and identified with the specific antibody for XRN1, phospho-IRF3, IRF3, and actin by WB assay. (B) *IFN-β* mRNA significantly increases in *XRN1* knockout A549 cells infected with Sendai virus. RNA was isolated from Sendai virus-infected control and *XRN1* knockout A549 cells at 4 h.p.i., and *IFN-β* levels were determined by RT-qPCR. Gene expression was calculated as fold change comparing mRNA expression of XRN1 KO cells against that of A549 control cells. ****, *P* < 0.0001. (C) Gene expression of innate immunity-related genes in *XRN1* knockout and control A549 cells after wild-type (WT) WSN or WSN DelNS1 infection. RNAs were isolated from WT WSN- or WSN DelNS1-infected A549 control and *XRN1* KO cells at 4 to 6 h.p.i. and evaluated with specific primers for *IFN-β*, *IFIT1*, *IFIT3*, and *ISG15* by RT-qPCR. Data represent means plus standard errors of the means of three independent experiments. Statistical significance was determined by a *t* test and indicated as follows: ***, *P* < 0.001; **, *P* < 0.01; *, *P* < 0.05. (D) The protein expression of p-IRF3 and IFIT3 in WT WSN- and WSN DelNS1-infected cells. The cellular and viral proteins were harvested from WT WSN- or WSN DelNS1-infected control and *XRN1* knockout A549 cells at 4 to 8 h.p.i. and identified with specific antibodies for XRN1, p-IRF3, IRF3, IFIT3, PB2, PB1, M1, NS1, and actin by WB assay.

The NS1 protein of IAV inhibits host IFN responses by binding to viral vRNA, RIG-I, MAVS, and TBK1, which are required for RIG-I activation (9, 44). As the above results indicated that IAV NS1 directly associate with XRN1 (Fig. 1), we investigated whether IAV NS1 is involved in XRN1-mediated suppression of the immune response. The A549 control and A549 XRN1 knockout cells were infected with Sendai virus. Phospho-IRF3 and the *IFN-β* mRNA were detected at 4 h.p.i. We found that XRN1 knockout led to increased expression of p-IRF3 and the mRNA of *IFN-β* compared with Sendai virus-infected A549 control cells (Fig. 7A and B). In a previous article, we reported that M-A14U substitution affects the splicing of M transcripts during viral replication and that M2 expression remains unchanged but M1 levels significantly decrease in DelNS1-M-A14U infection compared to wild-type WSN (WSN-WT) (45). We also determined that, despite lacking NS1, DelNS1-M-A14U viruses were able to replicate by modulating the alternative splicing of M mRNAs and that they cannot inhibit interferon expression in infected cells (45). Therefore, here we used the DelNS1-M-A14U viral model to study the innate immune response in the absence of the NS1 protein. We compared IFN-β-related host antiviral response during infection of WSN and WSN DelNS1-M-A14U (WSN DelNS1) viruses in the A549 control and A549 XRN1-KO cells. The mRNA expression of *IFN-β*, *IFIT1*, *IFIT3*, and *ISG15* antiviral genes was significantly higher in WSN DelNS1-infected XRN1 KO cells at 4 and 6 h.p.i. (Fig. 7C). Consistently, the protein levels of p-IRF3 and IFIT3 showed significantly higher expression in WSN DelNS1-infected XRN1 KO cells at 6 and 8 h.p.i. (Fig. 7D). These data indicate that the coexistence of IAV NS1 and host XRN1 causes synergistic effects and enhances the efficiency in suppressing the RIG-I-mediated IRF3 activity and IFN-β production.

## DISCUSSION

**FIG 8:**
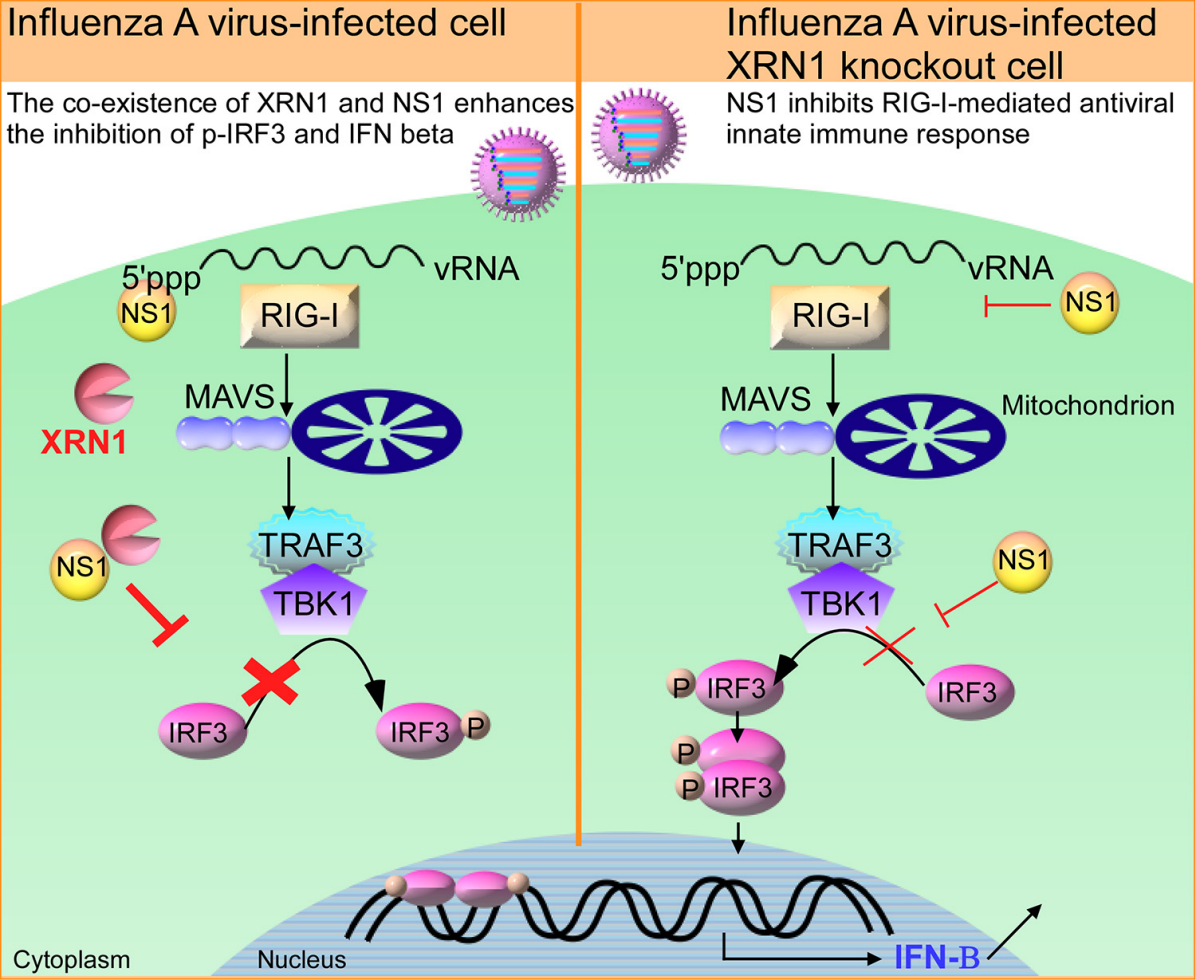
Schematic model of influenza A virus hijacks cellular XRN1 to suppress the host RIG-I-mediated innate immune response and to facilitate viral replication. The decay factor XRN1 of PBs contributes to influenza A virus replication and participates in the IAV vRNA-induced and RIG-I-mediated type I interferon response, resulting in the inhibition of IRF3 activity, IFN-β induction, and downstream ISG gene expression. The coexistence of IAV NS1 and host XRN1 enhances the inhibition of p-IRF3 and IFN-β production.

The NS1 protein of influenza virus plays an important role in viral replication, pathogenicity, and inhibition of innate immunity by multiple mechanisms. Notably, NS1 dramatically inhibits cellular gene expression and prevents the activation of key players in the IFN system through protein-protein interactions (44, 46). A schematic model is provided in Fig. 8. Our study uncovered a novel mechanism for influenza virus NS1 invasion of host cells involving its direct association with exoribonuclease XRN1 protein. The decay factor XRN1 contributes to influenza A virus replication and suppression of innate immune response, as this exoribonuclease inhibits IAV vRNA-induced type I interferon response, which results in the inhibition of IRF3 activation, IFN-β induction, and downstream ISG gene expression. In this study, we identified six components of PBs, including XRN1, EDC4, DCP1A, DCP2, DDX6, and RAP55, that could associate with NS1 without the use of an RNA intermediary (Fig. 1A). Then, we selected the exoribonuclease protein XRN1, which occupies a central position in the 5′-to-3′ mRNA decay complexes of PBs, for further analysis and confirmed the interaction between endogenous XRN1 and WSN NS1 without the intermediation of RNA (Fig. 1B and D). Furthermore, we further demonstrated that NS1 associates with the C-terminal region (aa 1174–1706) of XRN1 containing the C-terminal and EDC4-BM domains by overexpressing various truncated forms of XRN1 (Fig. 1C). Moreover, the assessment of NS1-XRN1 direct interaction was based on *in vitro* pulldown assays (Fig. 1D). We also observed that NS1 and XRN1 colocalized and accumulated with DCP1A in the PBs, which are distinct foci within the cytoplasm. This was easiest to observe at 6 and 8 h.p.i. in WSN-infected cells, as the NS1-XRN1 interaction was maintained between 4 and 8 h.p.i. (Fig. 2A and 1B). Moreover, the colocalization of endogenous XRN1 and FLAG-NS1 was detected in the cytoplasm of HEK293T cell through confocal microscopy (Fig. 2B), and the interaction without an RNA intermediary was detected using Co-IP (see Fig. S5A in the supplemental material). These results suggest that this interaction, aggregation, and accumulation between NS1 and XRN1 may provide advantages for the viral life cycle.

10.1128/mBio.00945-21.6 FIG S5 FLAG-NS1 can pull down the endogenous XRN1 without an RNA intermediary. (A) The FLAG-NS1 plasmid was transfected into the HEK293T cells and pulled down using anti-FLAG Dynabeads. We detected the cell lysates following RNase A treatment and WB assays with antibodies against endogenous XRN1 and FLAG, respectively. Download FIG S5, TIF file, 0.1 MB. Copyright © 2021 Liu et al. 2021 Liu et al. https://creativecommons.org/licenses/by/4.0/ This content is distributed under the terms of the Creative Commons Attribution 4.0 International license.

The data revealed that viral M1 and NS1 proteins decreased under knockdown of XRN1 (Fig. 3E) and knockout of XRN1 (Fig. 6A) at 8 h postinfection by using software normalized with the actin levels. In Fig. 5B, the decreased mRNA levels did not differ significantly after statistical analysis; however, the fold change of M mRNA decreased from 87.87- to 54.69-fold, and that of NS1 mRNA decreased from 81.96- to 67.17-fold in XRN1 KO cells at 8 h postinfection. Therefore, both the mRNA and protein levels of M1 and NS1 were consistently reduced in IAV-infected XRN1-depleted cells. The results suggest that the XRN1 may affect viral replication at 8 h postinfection. These two approaches consistently demonstrate the important role of XRN1 in supporting IAV replication. In this study, we demonstrate that WSN viral growth decreased in *XRN1* knockout and XRN1-deficient human lung cells. In contrast, the viral growth in WSN-infected HEK293T cells could be increased by the overexpression of FLAG-tagged XRN1 (Fig. 3). Furthermore, several mRNAs and proteins of the antiviral immune response, such as IFN-β, IFIT1, IFIT3, and ISG15, were observed to significantly increase in IAV-infected *XRN1* knockout A549 cells (Fig. 5). We have also measured *IFN* and *ISG* mRNA expression in A549 control cells and A549 XRN1 knockout cells, but after poly(I:C) treatment instead of WSN infection. We observed that the levels of *IFN-β* and *ISG* genes increased in XRN1 knockout A549 cells exposed to poly(I:C) treatment (see Fig. S3A in the supplemental material). As we know poly(I:C) is a synthetic analog of dsRNA that can act as agonist of RIG-I and MDA5, leading to IFN-β and ISG production. These results indicate that XRN1 can directly affect this innate immune response pathway under IAV infection. The H1N1 viruses tested (WSN, PR8, and CA04) showed similar infection levels in A549 control cells and A549 XRN1 KO cells at 8 h.p.i., based on viral NP protein expression (see Fig. S4A in the supplemental material). Moreover, we found that XRN1 KO cells infected with other IAV strains (see Fig. S4B in the supplemental material) also increased *IFN-β*, *IFIT1*, *IFIT3*, and *ISG15* mRNA expression. Here, we present evidence that the *IRF3* mRNA of XRN1 KO cells upon WSN, PR8, CA04, and HK4801 infection are similar to that of A549 control (Fig. 5C). The expressive proteins of IRF3 are equal in virus-infected XRN1 KO cells and A549 control cells, but p-IRF3 significantly increases in XRN1 KO cells after viral infection (Fig. 6A and Fig. S4A). Therefore, XRN1 affects IFN-β induction through the IRF3 phosphorylation, but not IRF3 protein expression. However, it remains unclear whether XRN1 affects the expression or activity of NF-κB, and this is worthy of further exploration. We therefore propose that XRN1 can be considered a host exoribonuclease factor for the positive regulation of influenza virus replication in human cells. This represents a strategy used by IAV NS1 to repress the host innate immune response through many pathways and allow efficient replication of virus (10, 44, 46).

10.1128/mBio.00945-21.4 FIG S3 The *IFN* and *ISG* mRNA expression in A549 control cells and A549 XRN1 KO cells after poly(I:C) treatment. (A) The *IFN* and *ISG* mRNA production in A549 control cells and A549 XRN1 knockout cells after poly(I:C) treatment instead of WSN infection. Data represent the means ± SD of three independent experiments. Statistical significance was determined by a *t* test. ***, *P* < 0.001; **, *P* < 0.01; *, *P* < 0.05; ns, not significant. Download FIG S3, TIF file, 0.3 MB. Copyright © 2021 Liu et al. 2021 Liu et al. https://creativecommons.org/licenses/by/4.0/ This content is distributed under the terms of the Creative Commons Attribution 4.0 International license.

10.1128/mBio.00945-21.5 FIG S4 The similar infection levels of H1N1 viruses in A549 control and A549 XRN1 KO cells. (A) The H1N1 viruses WSN, PR8, and CA04 showed similar infection levels in A549 control cells and A549 XRN1 KO cells at 8 h.p.i., based on viral NP protein expression. (B) The XRN1 KO cells infected with WSN, PR8, and CA04 strains also increased *IFN-β*, *IFIT1*, *IFIT3*, and *ISG15* mRNA expression. The fold changes in the amount of mRNA were calculated. Data represent the means ± SD of three independent experiments. Statistical significance was determined by a *t* test. ***, *P* < 0.001; **, *P* < 0.01; ns, not significant. Download FIG S4, TIF file, 0.8 MB. Copyright © 2021 Liu et al. 2021 Liu et al. https://creativecommons.org/licenses/by/4.0/ This content is distributed under the terms of the Creative Commons Attribution 4.0 International license.

Furthermore, we found that there was robust expression of IFN transcription factors and p-IRF3 in IAV-infected XRN1 KO cells (Fig. 6A). However, IRF3 phosphorylation by TBK1 was observed only in the IFN-producing pathways that use the adaptor protein MAVS to transduce signals from the cytosolic nucleic acid sensor RIG-I, which are activated by IAV viral RNA. Thus, we utilized purified IAV WSN vRNA to induce IRF3 activity in the type I IFN signaling pathway and found that XRN1 significantly affects the upstream of IFN-β transcription (Fig. 6B). RIG-I-like receptors are a type of intracellular pattern recognition receptor involved in the recognition of viruses by the innate immune system. RIG-I is a key sensor of influenza A virus infection, mediating the transcriptional induction of cellular innate interferon immune response upon detection of viral 5′ pppRNA. In addition to RIG-I, MDA5 is a significant contributor to the cellular defense against influenza A virus (36). Using siRNA against RIG-I and MDA5, we detected that the mRNA levels of *IFN-β* failed to be induced in IAV-infected XRN1 knockout cells. XRN1 can inhibit the IFN-β production through affecting the major RIG-I-mediated and minor MDA5-mediated IFN signaling pathways (Fig. 6C). XRN1 can inhibit p-IRF3, *IFN-β*, and the downstream *ISG* gene production. The NS1 of IAV may enhance the efficiency in XRN1-mediated suppression of IRF3 phosphorylation (Fig. 7).

Our findings reveal a previously unexplored mechanism of virus attack to host immune pathways whereby influenza virus NS1 directly interacts with the cellular exoribonuclease XRN1. IAV utilizes XRN1 to inhibit IRF3 activity, repress cellular *IFN-β* transcription, and then block IFN protein synthesis through RIG-I-mediated immune response. Our data indicate that host XRN1 has a strong impact on viral replication and the host innate immune responses to influenza A virus.

## MATERIALS AND METHODS

### Cell cultures and virus production.

Human lung adenocarcinoma cells (A549), human embryonic kidney cells (HEK293T), and Madin-Darby canine kidney (MDCK) cells were separately cultured in Dulbecco modified Eagle medium (DMEM) or minimal essential medium (MEM) containing 10% fetal bovine serum (FBS) and penicillin/streptomycin/glutamine (Gibco) at 37°C. H1N1 (A/WSN/1933), H1N1 (A/Puerto Rico/8/34), H1N1 (A/California/04/2009), and H3N2 (A/HK/4801/2014) were rescued using a DNA transfection system (15, 47, 48). Recombinant WSN viruses with deleted NS1 were prepared using eight pHW2000 plasmids containing mutant NS1-deleted gene segments, which were transfected into HEK293T cells and amplified in MDCK cells. The recombinant viruses were named DelNS1-M-A14U, as they adapted to gain an A14U substitution in the 3′-noncoding region of the M vRNA segment (45).

### Coimmunoprecipitation and Western blotting.

To map the interacting domains between XRN1 and NS1, the constructs of the various truncated forms of XRN1 and mutant NS1 were cotransfected into HEK293T cells using TransIT-LT1 reagent (Mirus) for 48 h. The cells were harvested and lysed with IP buffer (30 mM Tris-HCl [pH 7.4], 150 mM NaCl, 1% Triton X-100, and 1× proteinase inhibitor), the supernatant was treated with 10 μl of anti-GFP Dynabeads protein A (Invitrogen) added at 4°C for 12 h, and then 10 μg/ml RNase A was added at 30°C for 1 h. The coprecipitated proteins were collected with a magnet, followed by washing six times. The precipitated proteins were separated by 10% sodium dodecyl sulfate-polyacrylamide gel electrophoresis (SDS-PAGE) and subsequently identified using an anti-GFP antibody (diluted 1:5,000; Invitrogen), an anti-FLAG antibody (diluted 1:5,000; Sigma) or V5 antibody (diluted 1:5,000; Bio-Rad). Polyvinylidene difluoride (PVDF) membranes were analyzed by Odyssey scanner (LI-COR) using an IRDye 800CW or IRDye 680RD-linked secondary antibody.

### Protein purification and GST pulldown assay.

The pGEX-6P-XRN1-C-terminal region (aa 1174–1706) and pET32a-NS1 from various strains were transformed into BL21(DE3). Expression of the protein was induced by adding 1 mM or 0.1 mM isopropyl-β-d-thiogalactopyranoside (IPTG) at 16°C for 16 h. The protein expressed from lysed cells was suspended in IP buffer (30 mM Tris [pH 7.4], 150 mM NaCl, 1% Triton X-100) and sonicated with a SonicPrep ultrasonic homogenizer (PolyScience). GST-tagged-XRN1 (aa 1174–1706) and His-tagged NS1 proteins were purified with glutathione Sepharose 4B (GE Healthcare) and nickel-nitrilotriacetic acid (Ni-NTA) beads (Qiagen), respectively. The purity of recombinant proteins was determined by SDS-PAGE. Five micrograms of recombinant NS1 protein was mixed with GST-XRN1-bead or GST-bead (control) complexes at 4°C for 1 h. The targeted proteins were immunoprecipitated and analyzed by WB with an anti-GST antibody (diluted 1:1,000; ImmunoWay) and an anti-NS1 antibody (diluted 1:1,000; National University of Singapore [NUS]).

### Immunofluorescence microscopic analysis.

A549 cells grown on Millicell EZ glass slide (Millipore) at 80% confluence were infected with WSN at an MOI of 2 for 4 to 8 h.p.i. or were transfected with 2 μg of the WSN NS1 clone. The cells were fixed in phosphate-buffered saline (PBS) containing 4% formaldehyde, permeated with 0.3% Triton X-100, blocked with 5% normal donkey serum for 1 h at 25°C, and then stained with anti-XRN1 (diluted 1:20; Santa Cruz), anti-WSN NS1 (diluted 1:100; NUS, anti-DCP1A (diluted 1:20; Santa Cruz), or anti-FLAG (diluted 1:100; Sigma) antibodies for 2 h at 37°C. Subsequently, the cells were stained with fluorescein isothiocyanate (FITC)-conjugated goat anti-mouse IgG (diluted 1:200; green, Invitrogen), goat anti-rabbit IgG (diluted 1:200; red, Invitrogen), or donkey anti-goat IgG (diluted 1:200; purple, Invitrogen) for 1 h at 25°C. The cells were washed three times with PBS and mounted in Vectashield antifade mounting medium with 4′,6′-diamidino-2-phenylindole (DAPI) (Vector Laboratories). Confocal images were obtained with a confocal laser-scanning microscope (Zeiss; LSM 700).

### Generation of A549 CRISPR-Cas9 genetically modified cell lines.

The *XRN1* genes in A549 cells were inactivated using the CRISPR-Cas9 system using previously described methods (43, 49). The *XRN1* target sequence GTATCCCTGTCTCAGCGAAG, along with plasmid vector pSpCas9(BB)-2A-Puro (PX459) v2.0 (Genscript), which drives expression of the Streptococcus pyogenes Cas9, a puromycin resistance cassette, and the chimeric guide RNA was introduced into mammalian cells. A549 cells (7 × 10^4^ cells/well) seeded in 24-well plates were transfected with the recombinant plasmids using Lipofectamine 3000 (Life Technologies) for 1 day, after which 1 mg/ml of puromycin was added for a 3-day selection. Then, the cells were reseeded in 96-well plates (1 cell/well) without puromycin treatment. After 21 days, total protein was prepared from individual colonies, and the absence of proteins was confirmed by sequencing *XRN1* genomic DNA and Western blotting. The XRN1 knockout cells were generated from a single clone.

### Generation of XRN1-deficient cells.

A549 cells (1.5 × 10^5^ cells/well) were transfected with 100 μM *XRN1* small interfering RNA (siRNA) and AllStars negative-control siRNA (Qiagen) using Lipofectamine 3000 (Life Technologies). The *XRN1* siRNA-1 sequence is 5′-CAGGUCGUAAAUAUCAAAUAA-3′ (Qiagen), and the *XRN1* siRNA-2 sequence is 5′-GGGAUCUGGAAAGAUGCAAUACUUU-3′ (Invitrogen). At 48 h posttransfection with siRNA, cells were reseeded at 1 × 10^5^ cells/well in a 24-well plate and were incubated for 24 h for further WSN infection and plaque assay.

### Viral growth kinetics and plaque assay.

A549 control cells or A549 *XRN1* knockout cells were seeded in 24-well plates (1 × 10^5^ cells/well) and, after 24 h, were washed and infected with viruses at an MOI of 0.01. Following viral absorption at 37°C for 1 h, the infected cells were washed twice with PBS and subsequently covered with MEM containing 1 μg/ml tosylsulfonyl phenylalanyl chloromethyl ketone (TPCK) trypsin and incubated at 37°C. Supernatants were collected at different time points, and the titers of the virus were determined by plaque assay in MDCK cells. Monolayer MDCK cells in six-well plates (6 × 10^5^ cells/well) were washed and infected with virus by performing 10-fold serial dilutions. After 1-h adsorption, the cells were washed twice with PBS and then overlaid with MEM containing 1 μg/ml TPCK trypsin and 0.3% agarose gel at 37°C for 48 h. The plates were fixed in 10% formaldehyde and stained with 1% crystal violet. The viral titers are presented as the number of PFU per milliliter.

### RNA extraction and qRT-PCR.

Total RNA was extracted from mock-infected and IAV-infected A549 control cells or A549 *XRN1* knockout cells using RNAzol RT (Molecular Research Center), per the manufacturer’s instructions. RNAs were converted into first-strand cDNAs using a PrimeScript RT reagent kit (TaKaRa) with oligo(dT) reverse primers. All PCRs were performed using specific primers (see Table S1 in the supplemental material), which were purchased from Integrated DNA Technologies (IDT). The qPCR analysis was performed using SYBR green reagents and the LightCycler 480 instrument (Roche).

10.1128/mBio.00945-21.1 TABLE S1 Sequences of primers used for RT-qPCR. Download Table S1, DOCX file, 0.01 MB. Copyright © 2021 Liu et al. 2021 Liu et al. https://creativecommons.org/licenses/by/4.0/ This content is distributed under the terms of the Creative Commons Attribution 4.0 International license.

### MTT assay.

A549 cells (2× 10^3^ cells/well) were seeded in 96-well plates for 24 h, and then the cells were treated with various concentrations of pAp (1 to 6 mM) (catalog no. A5763; Sigma). The viability of pAp-treated A549 cells was validated by MTT assay (Abcam) in accordance with the manufacturer’s instructions.
