# Supplementary figures and images for: Cellular 5′-3′ mRNA Exoribonuclease XRN1 Inhibits Interferon Beta Activation and Facilitates Influenza A Virus Replication
Source: mBio. 2021 Jul 27;12(4):e00945-21. doi: 10.1128/mBio.00945-21 (PMC8406323; doi:10.1128/mBio.00945-21)

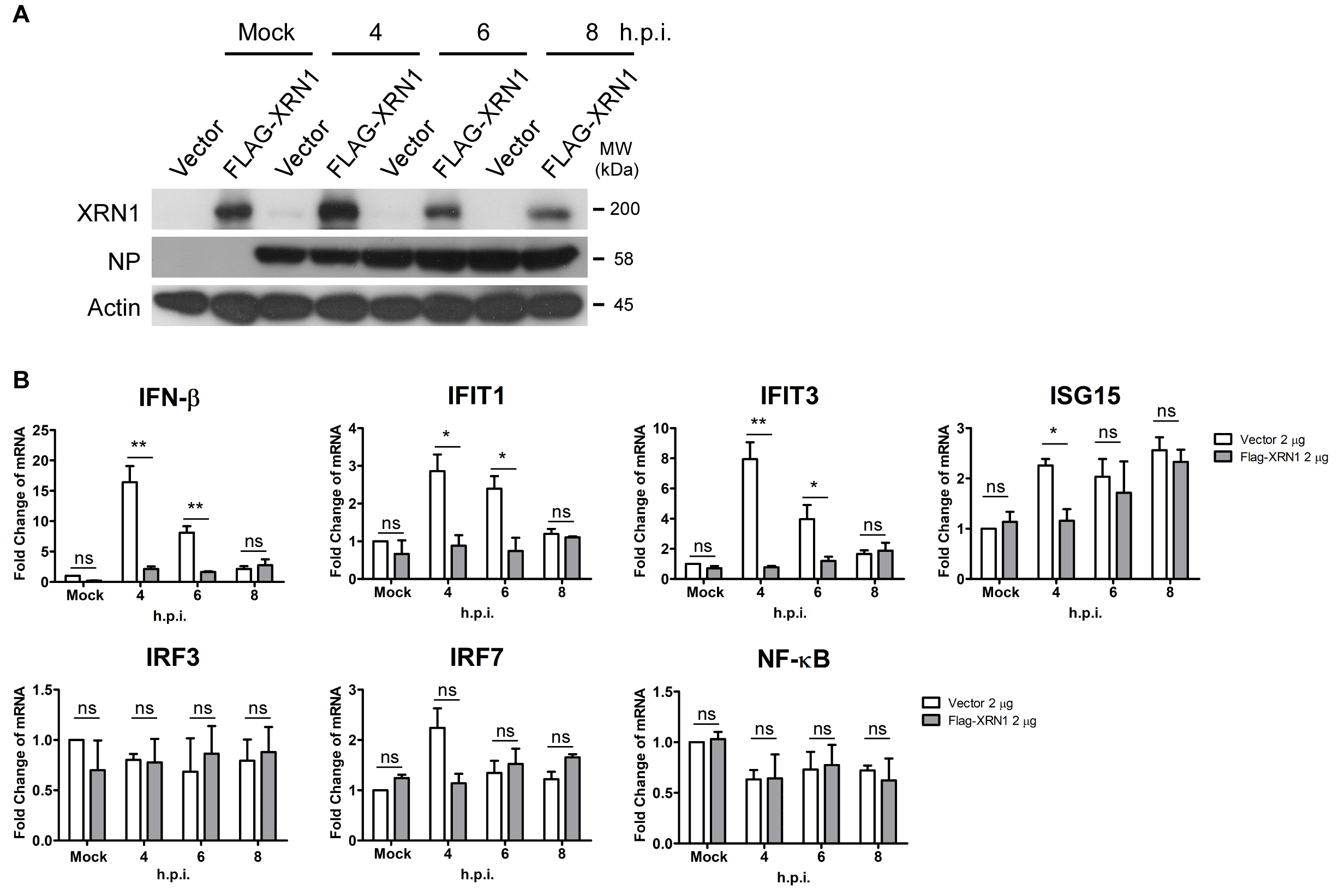

Supplement: FIG S2 [file mbio.00945-21-sf002.tif]

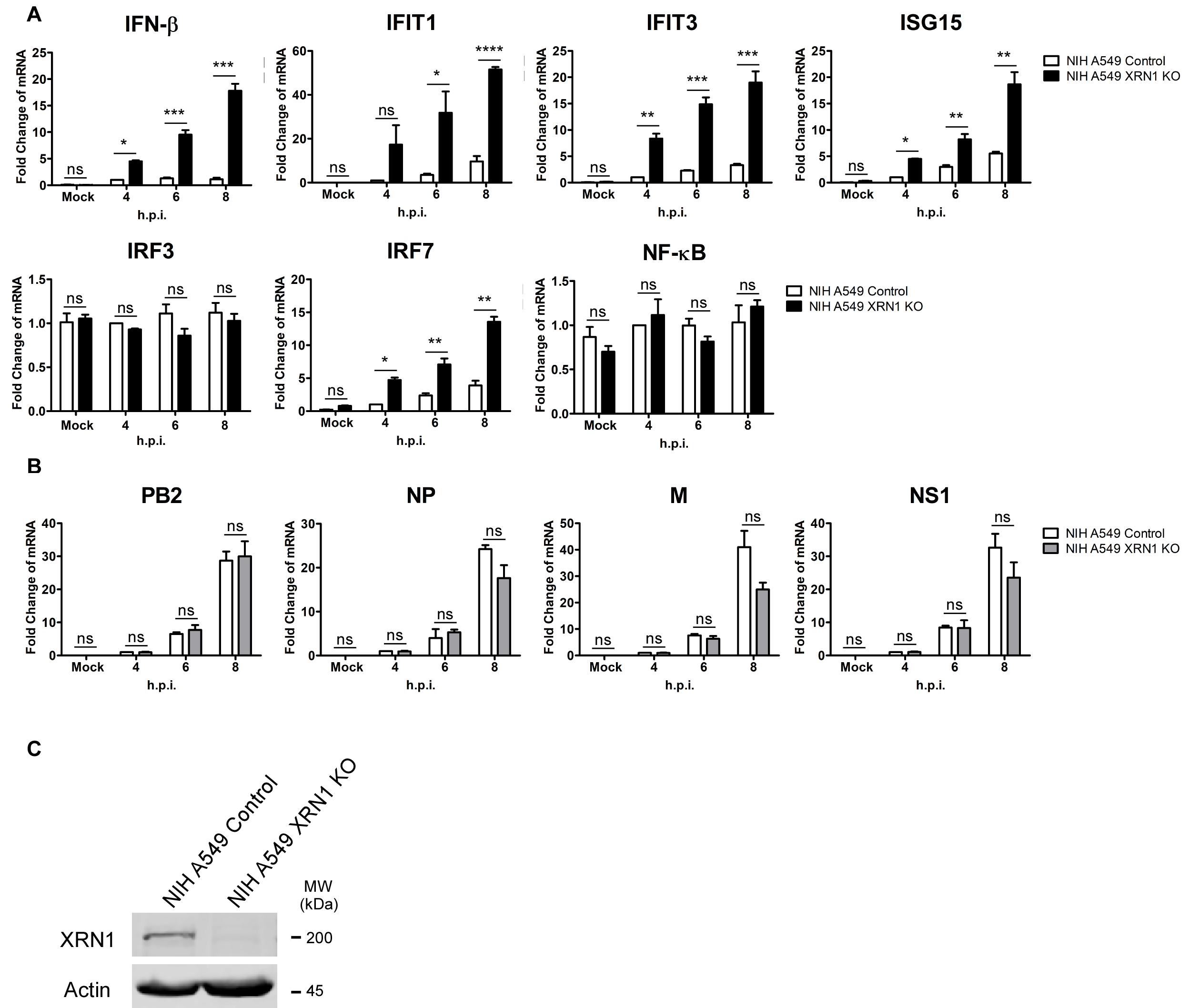

Supplement: FIG S1 [file mbio.00945-21-sf001.tif]

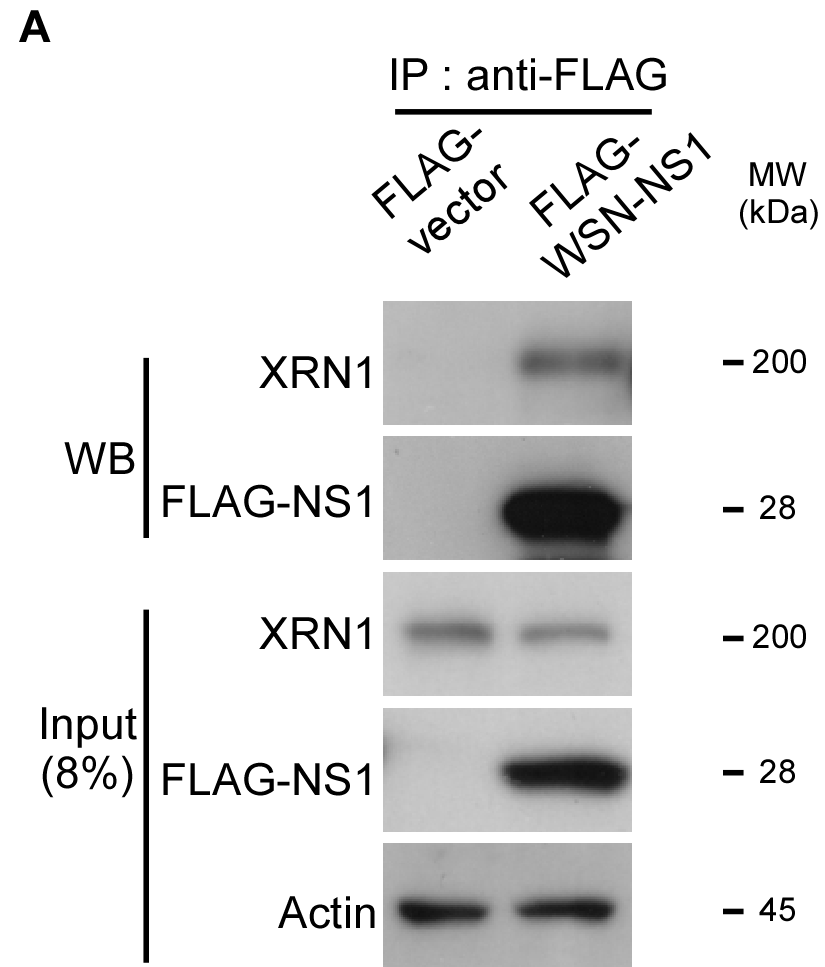

Supplement: FIG S5 [file mbio.00945-21-sf005.tif]

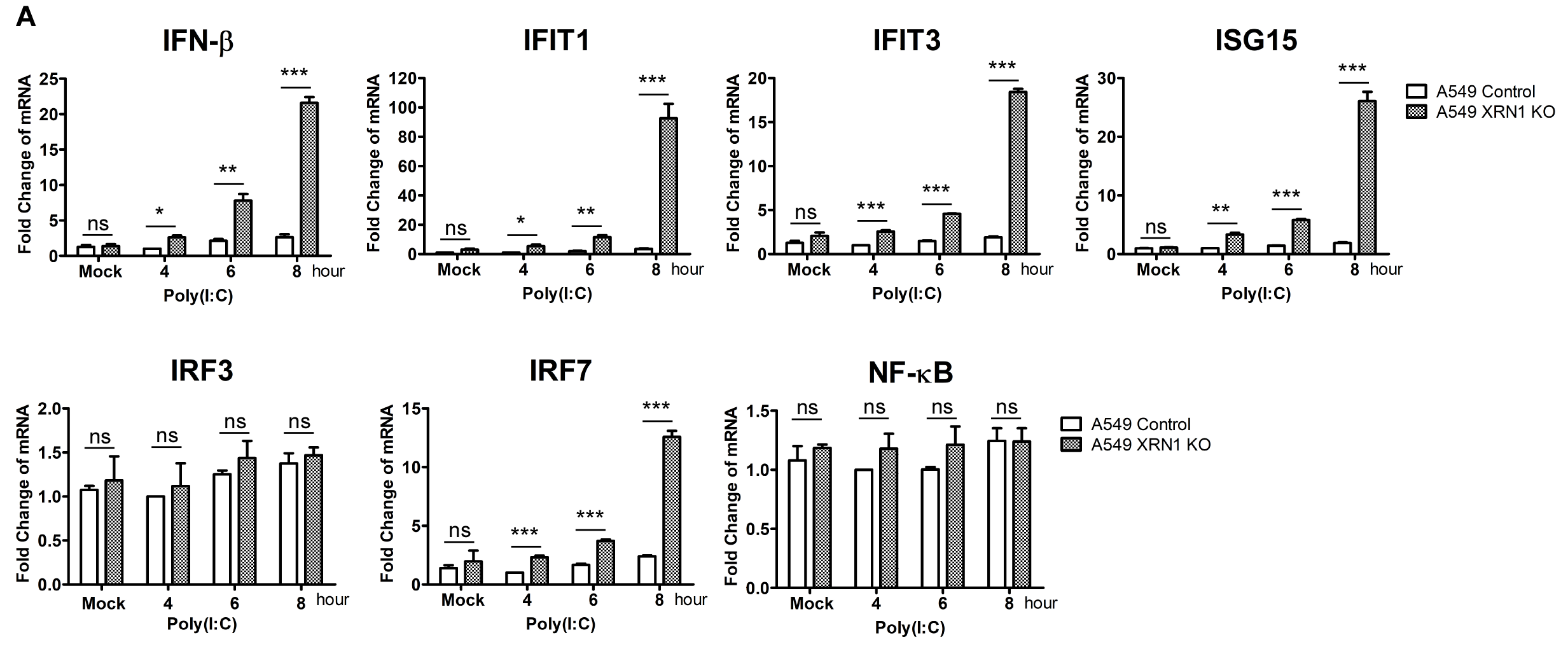

Supplement: FIG S3 [file mbio.00945-21-sf003.tif]

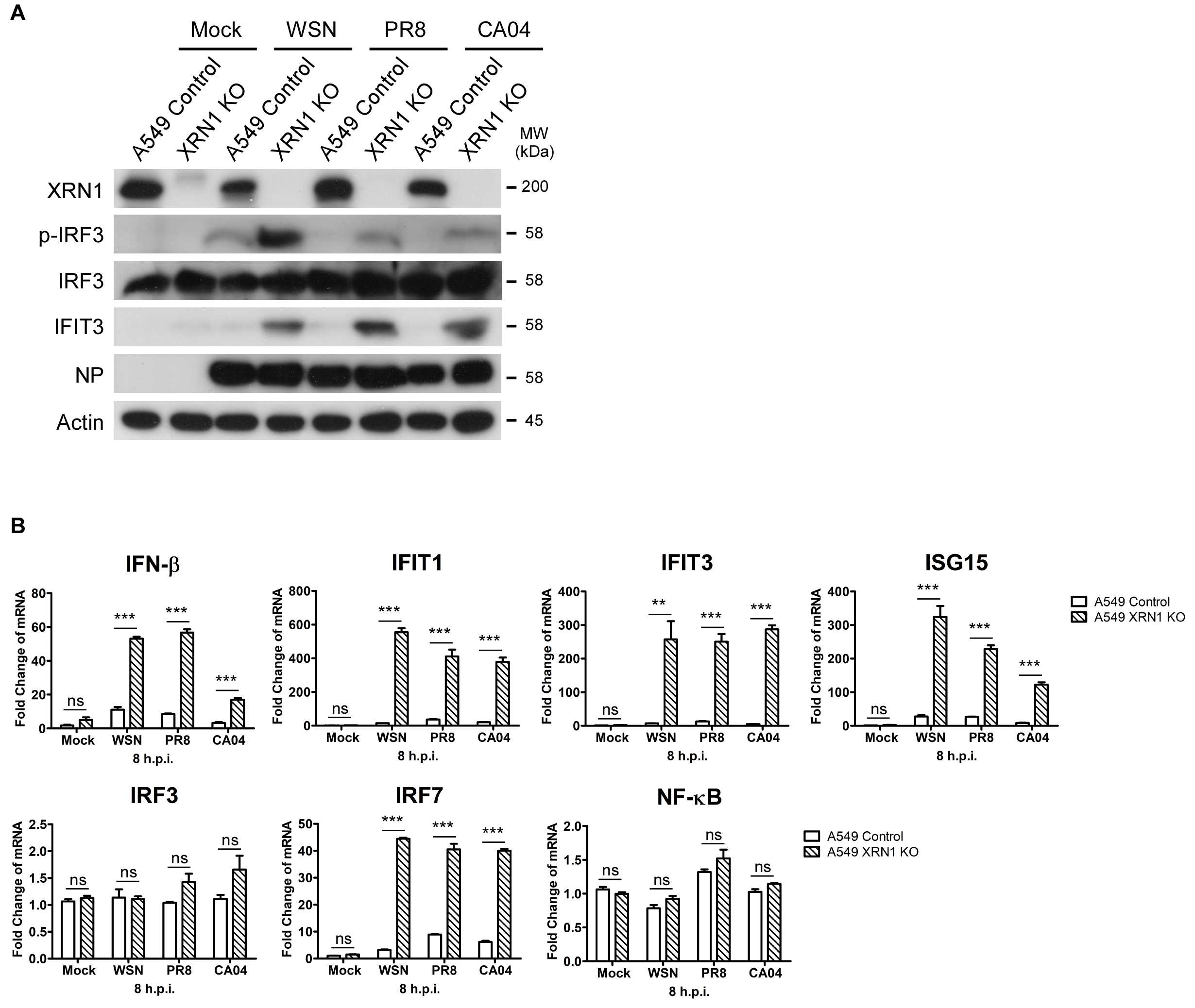

Supplement: FIG S4 [file mbio.00945-21-sf004.tif]
