# Supplementary material for: Cellular 5′-3′ mRNA Exoribonuclease XRN1 Inhibits Interferon Beta Activation and Facilitates Influenza A Virus Replication
Source: mBio. 2021 Jul 27;12(4):e00945-21. doi: 10.1128/mBio.00945-21 (PMC8406323; doi:10.1128/mBio.00945-21)
Supplement: TABLE S1 [file mbio.00945-21-st001.docx]

**Table S1.** The sequence of primers was used for RT-qPCR

| Name | Sequence |
| --- | --- |
| IFN-β-For | 5'-GGCAATTGAATGGGAGGCT-3' |
| IFN-β-Rev | 5'-GGCGTCCTCCTTCTGGAACT-3' |
| IFIT1-For | 5'-TCCAGGGCTTCATTCATAT-3' |
| IFIT1-Rev | 5'-TTCGGAGAAAGGCATTAGA-3' |
| IFIT3-For | 5'-AGAAAAGGTGACCTAGACAAAGC -3' |
| IFIT3-Rev | 5'-CCTTGTAGCAGCACCCAATCT-3' |
| ISG15-For | 5'-CGCAGATCACCCAGAAGATCG-3' |
| ISG15-Rev | 5'-TTCGTCGCATTTGTCCACCA-3' |
| IRF3-For | 5'-CAGCTTGGACAATCCCACTC-3' |
| IRF3-Rev | 5'-GTCACCTCGAACTCCCACTC-3' |
| IRF7-For | 5'-GCAGCGTGAGGGTGTGTCTT-3' |
| IRF7-Rev | 5'-GCTCCATAAGGAAGCACTCGAT-3' |
| NF-κB-For | 5'-CCTGGATGACTCTTGGGAAA-3' |
| NF-κB-Rev | 5'-TCAGCCAGCTGTTTCATGTC-3' |
| WSN-PB2-For | 5'-GCGAATCAGCGATTGAACCC-3' |
| WSN-PB2-Rev | 5'-TCCGCGCTGGAATACTCATC-3' |
| WSN-NP-For | 5'-GGTGAGAATGGACGGAGAAC-3' |
| WSN-NP-Rev | 5'-CCGGCTCTCTCTCACTTGAT-3' |
| WSN-M-For | 5'-CGGTCTCATAGGCAAATGGT-3' |
| WSN-M-Rev | 5'-CAATATCCATGGCCTCTGCT-3' |
| WSN-NS1-For | 5'-CAGCACTCTTGGTCTGGACA-3' |
| WSN-NS1-Rev | 5'-ACCAGTGCCTTGACATTTCC-3' |
